# Draft Whole-Genome Shotgun Sequence of Streptomyces sp. Strain ETH9427

**DOI:** 10.1128/MRA.01197-18

**Published:** 2018-09-27

**Authors:** Annabelle Thibessard, Cláudia M. Vicente, Claire Bertrand, Bertrand Aigle, Pierre Leblond

**Affiliations:** a Université de Lorraine, Inra, DynAMic, Nancy, France; Louisiana State University

## Abstract

The draft genome of Streptomyces sp. strain ETH9427 was sequenced and assembled into three large scaffolds, a 7.745-Mb linear chromosome with terminal inverted repeats of 201 kb and two probable extrachromosomal elements. Thirty-two biosynthetic gene clusters (BGCs) were identified, out of which four are duplicated in the terminal inverted repeats.

## Abstract (precis)

The draft genome of Streptomyces sp. strain ETH9427 was sequenced and assembled into three large scaffolds, a 7.745-Mb linear chromosome with terminal inverted repeats of 201 kb and two probable extrachromosomal elements.

## ANNOUNCEMENT

We report here the draft genome sequence of Streptomyces sp. ETH9427, a strain initially reported as Streptomyces ambofaciens ETH9427 (1). The strain was obtained from Ralf Hütter (1) and has been maintained at the DynAMic lab since then. The sequencing project was aimed at unveiling the biosynthetic gene clusters (BGCs) and comparing the BGC content of the genomes of closely related Streptomyces isolates and comparing their genome organizations.

After growth in liquid Hickey-Tresner medium (2) at 30°C for 30 h and DNA purification (2), two libraries, a mate pair (∼8 kb) and a paired-end (∼300 bp) library, were created (MiSeq reagent kit version 3; 600 cycles), from which 3,329,310 and 405,558 reads (average size, 301 bp), respectively, were generated using a Genome Analyzer (Illumina). The reads were assembled with Velvet version 1.2.10 (default settings, option -scaffolding, “yes”; *k*-mer, 95) into a total of 68 scaffolds (*N*_50_, 1.33 Mb) comprising 739 contigs (*N*_50_, 19,192 bp), for a total estimated genome size of 7.8 Mb (genome coverage, 144×). Contig order was validated by reference to closely related strains using the Artemis Comparison Tool (3). Coding sequence prediction and annotation were performed using the NCBI Prokaryotic Genome Annotation Pipeline (4).

The genome sequence of Streptomyces sp. strain ET9427 consists of a linear chromosome of 7,745,357 bp with long terminal inverted repeats of 201,878 bp and an average GC content of 72.07%. Six rRNA operons, 68 tRNAs, and 7,140 protein-coding genes were found on the chromosome. The following two probable extrachromosomal elements were characterized: (i) pETH1, a linear plasmid of 106,771 bp (GC content, 71.7%) having, at the chromosome’s end, typical palindromic telomere sequences and possessing two complete CRISPR loci, as predicted by CRISPRfinder (5; see also http://crispr.i2bc.paris-saclay.fr/); and (ii) pETH2, a 125,903-bp plasmid (GC content, 71.3%), which was also predicted to be linear. The PHAge Search Tool (PHAST) (6) predicted that this latter molecule contains or constitutes a whole bacteriophage.

Assessment of the phylogenetic relationship of the species using average nucleotide identity (ANI) values (7; see also http://enve-omics.ce.gatech.edu/ani) determined that the closest species whose genome is available is Streptomyces sp. strain 4F with an ANIb value of 94% (GenBank accession number CP013142). Despite its first assignment (1), the ANIb value (∼81%) was too low to support its assignment to the species S. ambofaciens; it does, however, support the organism being renamed Streptomyces sp. The isolates Streptomyces sp. strain ETH9427, Streptomyces sp. strain 4F, and S. ambofaciens strain ATCC 23877 (8) constitute, however, a group of related strains, since the divergence of their 16S rRNA genes does not exceed 1%.

A preliminary analysis of the Streptomyces sp. strain ETH9427 sequence using antiSMASH (9, 10) allowed the identification of a total of 32 BGCs, out of which 4 were found to be duplicates since they lay in the terminal inverted repeats. Strains Streptomyces sp. ETH9427, Streptomyces sp. 4F, and S. ambofaciens ATCC 23877 shared nine BGCs, while three additional BGCs were conserved between Streptomyces sp. strains ETH9427 and 4F, confirming the phylogenetic relationship inferred by the ANIb analysis.

### Data availability.

The whole-genome sequence reported here has been deposited in GenBank under the accession numbers CP029624, CP029625, and CP029626. Raw sequence reads (Illumina) have been deposited in the NCBI Sequence Read Archive under the accession number SRP158364.
